# Effect of melatonin versus vitamin D as antioxidant and Hepatoprotective agents in STZ-induced diabetic rats

**DOI:** 10.1186/s40200-017-0322-6

**Published:** 2017-09-29

**Authors:** Abdulmonim A. Alqasim, Essam Eldin M. Noureldin, Sami H. Hammadi, Ghada E. Esheba

**Affiliations:** 1 Department of Physiology, College of Medicine, Umm Alqura University, Makkah, Saudi Arabia; 2 Department of Biochemistry, College of Medicine, Umm Alqura University, Makkah, Saudi Arabia; 3 Department of Internal Medicine, College of Medicine, Umm Alqura university, Makkah, Saudi Arabia; 4 Department of Pathology, College of Medicine, Umm Alqura University, Makkah, Saudi Arabia

**Keywords:** Diabetes, Liver, Melatonin, Vitamin D, Oxidative stress

## Abstract

**Background:**

Diabetes mellitus (DM) is a serious chronic disease, with multiple complications including hepatopathy associated with imbalance of the oxidative status. The purpose of this study is to observe possible protective effects of vitamin-D and melatonin on glucose profile, antioxidant-oxidant status, lipid peroxidation, and histopathological protection of the liver in streptozotocin-induced diabetic rats.

**Methods:**

Eighty three male albino rats were divided into nine groups as follows: **G1** (*n* = 10) Normal control rats; **G2** (*n* = 8) were normal rats treated with melatonin only; **G3** (*n* = 10) were normal rats treated with vitamin D only; **G4** (*n* = 9) were diabetic rats, which received no medications; **G5** (*n* = 8) were diabetic rat treated with insulin only; **G6** (*n* = 10) were diabetic rats treated with melatonin only; **G7** (*n* = 9) were diabetic rats treated with melatonin and insulin; **G8** (*n* = 9) were diabetic rats treated with vitamin D only; **G9** (*n* = 10) were diabetic rats treated with vitamin D and insulin. Two months post treatment, blood was collected to measure: Fasting blood sugar (FBS), glycosylated hemoglobin (HbA1c), fructosamine (FA), total antioxidant capacity (TAC), malondialdahyde (MDA). livers were isolated for histopathological study.

**Results:**

As compared to normal rats, our results demonstrate that glucose, fructosamine and HbA1c levels is increased in diabetic groups and declined to lesser levels in treated groups. TAC level of diabetic rats is not significantly changed. Vitamin D administration significantly increased TAC while it is not changed with melatonin either in treated or non-treated groups. The liver of diabetic rats shows only mild focal microvesicular fatty degeneration. The liver of diabetic rats treated with insulin shows degeneration of cell edema in the stroma. The liver of diabetic rats treated with melatonin with or without insulin, exhibited marked improvement. The liver of diabetic rats treated with vitamin D with or without insulin, shows degeneration of cells and edema in the stroma.

**Conclusion:**

Our results demonstrated the beneficial antioxidant effect of vitamin D administration to normal and diabetic rats as compared to melatonin. Nevertheless, melatonin still shows more therapeutic effect on liver cell injury induced by induction of diabetes.

## Background

Diabetes mellitus (DM) is a serious chronic disease, which incidence is globally increasing and considered as an epidemic [1]. The prevalence of diabetes is mainly due to an increased prevalence of type 2 diabetes (T2D). The incidence of type 1 diabetes (T1D) is also increasing in parallel to that of T2D worldwide with major health and socio economic impacts [2].

Liver plays vital roles in carbohydrate, lipid and protein synthesis and metabolism [3]. Studies have demonstrated that DM can lead to several liver defects, such as non-alcoholic fatty liver disease (NAFLD) [4], abnormal glycogen accumulation, cirrhosis and liver carcinomas [5–7].The underlying mechanisms that accelerate hepatopathy in patients with diabetes is not fully understood. Several mechanisms have been postulated to explain the damaging effect of DM on the liver. Both hyperglycemia-induced oxidative stress and hyperglycemia-induced inflammatory responses act as hepatocellular damaging factors [8]. Oxidative stress is defined as an imbalance in the oxidant-to-antioxidant ratio, causing the generation of free radicals [9]. Activated Kupffer cells production of free radicals is a central factor to hepatic injuries [10]. Excessive production of reactive oxygen species (ROS) results in various detrimental events, such as irreversible oxidative modification of lipids, proteins and carbohydrates [11]. ROS could activate the release of inflammatory mediators leading to induction of adhesion molecules and infiltration of leukocytes. Moreover, ROS could provoke apoptosis in hepatocytes causing massive destruction of liver tissue [12]. Diabetes is associated with reduced level of glutathione causing accumulation of oxidative stress product such as lipid peroxidation, which subsequently causes substantial increase in malondialdahyde, a marker for oxidative stress [13].

Various antioxidants have been demonstrated to have hepatoprotective effect, such as *ginkgo biloba* extract, resveratrol, 17β-estradiol, arjunolic acid, α-lipoic acid, L-cysteine and melatonin through anti-oxidative, anti-inflammatory, anti-apoptotic and/or antidiabetic properties [8].

Melatonin is a powerful antioxidant and the only currently available molecule known to block all aspects of the “devil’s triangle” [14]. This pineal gland’s generated hormone has been shown to play various regulatory roles such as regulation of circadian rhythm, sexual behavior, immune function, energy metabolism, regulation of the cardiovascular and the reproductive system. Melatonin also demonstrated a potent antioxidant capability and possessed protective properties against oxidative stress [3]. It has been shown that melatonin ameliorates oxidative damage in hyperglycemia-induced liver injury [13]. Oral melatonin administration reduces liver steatosis and mitochondria dysfunction in diabetic rats [15]. Melatonin administration partially reduced liver injury in streptozotocin-induced diabetic rats [5]. However, its protective effect on DM-mediated liver dysfunction need further investigation. The role of vitamin D in the pathogenesis of many diseases including DM is growing. The link between vitamin D and various DM-associated disorders such as renopathy, retinopathy and vasculopathy have been reported [16]. However, the available studies on its beneficial effects on DM-mediated liver dysfunction are limited and controversial [17, 18]. The purpose of this study is to observe possible protective effects of vitamin-D and melatonin on glucose profile, antioxidant-oxidant status, lipid peroxidation, and histopathological protection of the liver in streptozotocin-induced diabetic rats. Positive observation from these treatments will open new window for better treatment of this chronic disease.

## Methods

### Induction of DM

Diabetes mellitus in rats was induced by intra-peritoneal administration of nicotinamide (230 mg/kg), 15 min prior to the single dose of streptozotocin (STZ) (65 mg/kg, i.p.) [19]. Control animals were received an equal volume of saline. The STZ was dissolved in saline with a sodium citrate buffer, pH 4.0. The blood glucose levels (by using standard diagnostic kits) were recorded to monitor the degree of diabetes. Confirmation of induction of diabetes was made by measuring blood glucose level prior to further treatment. Rats with established hyperglycemia were used in the study.

### Groups and treatments

Eighty three male albino rats (200–250 g) were divided into nine groups as follows: **G1** (*n* = 10) Normal fed diet rats were served as control, which received no medications; **G2** (*n* = 8) were normal rats treated orally with melatonin only (0.3 mg/kg); **G3** (*n* = 10) were normal rats treated orally with vitamin D only (40 mg/kg); **G4** (*n* = 9) were diabetic rats, which received no medications; **G5** (*n* = 8) were diabetic rat treated with insulin only; **G6** (*n* = 10) were diabetic rats treated orally with melatonin (0.3 mg/kg) only; **G7** (*n* = 9) were diabetic rats treated orally with melatonin (0.3 mg/kg) and insulin; **G8** (*n* = 9) were diabetic rats treated orally with vitamin D (40 mg/kg) only; **G9** (*n* = 10) were diabetic rats treated orally with vitamin D (40 mg/kg) and insulin. The duration of the treatment was for 8 weeks and the dose of insulin was calculated according to the weight of each rat and the level of blood glucose.

### Biochemical measurements

Two months post treatment, rats were sacrificed and blood was collected for biochemical measurement. Fasting blood sugar (FBS), glycosylated hemoglobin (HbA1c), fructosamine (FA), total antioxidant capacity (TAC), malondialdahyde (MDA) were determined using the standard procedures and available commercial kits in a fully automated system. All assays were done by following the recommended procedures for instrument operation, calibration, quality control, and assay guidelines.

Blood samples were drawn in ethylene diamine tetra acetic acid (EDTA)-containing vacationer tubes for measuring glycosylated hemoglobin (HbA1c) in the same day. For serum experiments, samples were obtained following collection of blood in plain tubes and left for 30 min, then centrifuged for 15 min at 3000 rpm. Aliquots (1 ml) were separated in different Eppendorf tubes for the determination of the blood level of FBS, FA, TAC and MDA. Serum samples were then kept in −80 °C for later analysis.

Measurement of TAC, was performed by using rat TAC ELISA Kit from MyBioSource, Inc. The combined antioxidant activities of all vitamins, proteins, lipids, glutathione, uric acid, and others were assessed. MDA was measured by using rat TBARS ELISA assay kit (MyBioSource, Inc) for assessment of lipid peroxidation in rat serum. The assay is based on the measurement of the byproduct of the reaction between MDA and thiobarbituric acid under high temperature (90–100 °C). The byproduct was measured calorimetrically at 530–540 nm and the concentration of MDA was expressed in μM. Measurement of fructosamine was performed by using the fructosamine reagent set (POINTE SCIENTIFIC, Inc., Canton, Michigan, USA). Measurements of FBS and HbA1c were done using the standard procedures and available commercial kits in a fully automated system (COBAS integra 400 plus). The instrument was calibrated using calibrator for automated systems (Roche Diagnostics). Sensitivity, linearity and precision of the used scientific, commercial kits were evaluated by the manufacturers. The % CV of all kits was less than 5%.

### Histopathological examination

The rats were sacrificed and the livers were taken for histopathological examination. Tissue sections were immediately fixed in formalin (10%) and the specimens were then immersed in a series of ethanol (alcohol) solutions of increasing concentration until pure, water-free alcohol is reached. The specimens were then immersed in xylene to completely displace ethanol. The tissues were then embedded in paraffin wax to form the “blocks”, which were then clamped into a microtome for section cutting. The sections were then attached to microscope slides. The embedding process were then reversed in order to get the paraffin wax out of the tissue and allow water-soluble dyes to penetrate the sections. The slides were “deparaffinized” by running them through alcohols to water. The staining was done using hematoxylin and eosin (H & E). The stained section on the slides were then covered with a thin pieces of glass cover.

### Statistical analysis

Descriptive statistics and one way ANOVA were used to compare the concentration of the metabolic parameters between the nine groups. *P* value of <0.05 was considered as statistically significant. All statistical methods were performed using SPSS for windows (version 20, SPSS Inc.).

## Results

### Biochemical findings

**Fig. 1:**
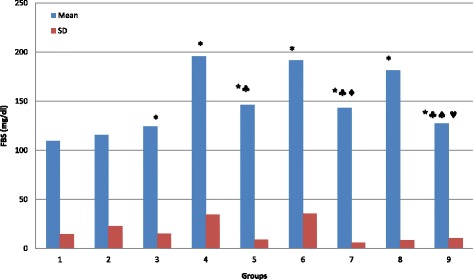
Effect of vitamin D and melatonin on the level of FBS on diabetic rats

**Fig. 2:**
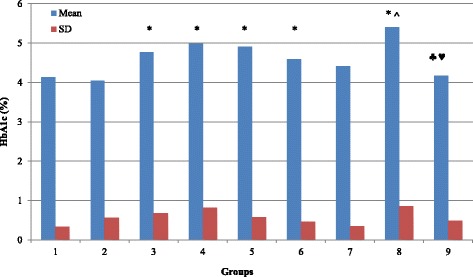
Effect of vitamin D and melatonin on the % of HbA1c on diabetic rats

**Fig. 3:**
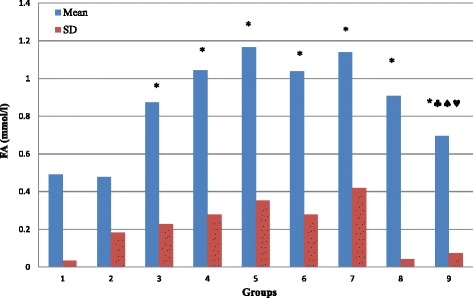
Effect of vitamin D and melatonin on the level of FA on diabetic rats

**Fig. 4:**
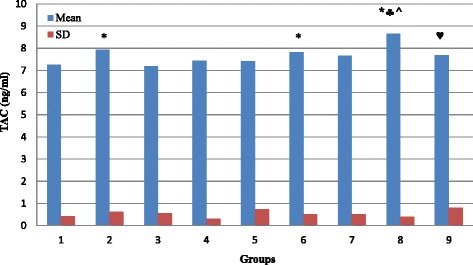
Effect of vitamin D and melatonin on the level of TAC on diabetic rats

**Fig. 5:**
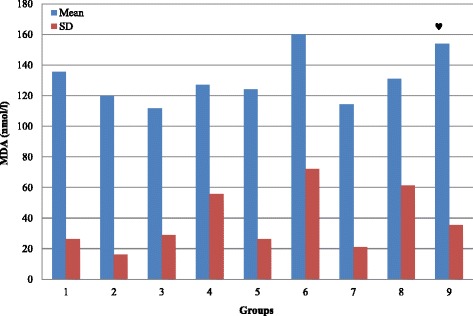
Effect of vitamin D and melatonin on the level of MDA on diabetic rats

As compared to normal rats (G1), our results demonstrate that glucose and fructosamine levels is significantly increased in G3, G4, G5, G6, G7, G8 and G9 (*P* < 0.05) with no significant change in G2 (Table 1; Figs. 1 and 3; *P* > 0.05). Figure 2 demonstrate that the percentage of HbA1c is significantly increased in G3, G4, G5, G6 and G8 (*P* < 0.05) with no significant changes in G2, G7 and G9 (*P* > 0.05). TAC is significantly increased in G2, G6 and G8 (*P* < 0.05), and showed no significant changes in G3, G4, G5, G7, and G9 (*P* > 0.05; Fig. 4). MDA showed non-significant changes in all groups. However, there is a decrease in its level in G3 and G7 but it is non-significant (*P* > 0.05; Fig. 5).

**Table 1:** Summary of the effect of vitamin D and melatonin on the level of FBS, HbA1c, FA, TAC and MDA on diabetic rats

Group	G1	G2	G3	G4	G5	G6	G7	G8	G9
FBS (mg/dl)	109.6	115.5	124.2	195.78	146.38	191.4	143	181.6	127.43
SD	14.569	23.016	15.076	34.72	9.133	35.635	5.874	8.649	10.309
Significance			*	*	* ♣	*	* ♣♦	*	*♣♠♥
HbA1c (%)	4.137	4.0375	4.76	4.9778	4.9	4.59	4.4111	5.4	4.1714
SD	0.33751	0.56553	0.6802	0.81972	0.5757	0.46536	0.35158	0.86023	0.4855
Significance			*	*	*	*		* ^	♣♥
FA (mmol/l)	0.491	0.479	0.872	1.043	1.166	1.037	1.139	0.908	0.694
SD	0.0348	0.1817	0.2271	0.2775	0.353	0.2796	0.4198	0.0432	0.0737
Significance			*	*	*	*	*	*	*♣♠♥
TAC (ng/ml)	7.249	7.924	7.188	7.433	7.426	7.813	7.671	8.65	7.693
SD	0.4189	0.6181	0.5495	0.3024	0.7472	0.5195	0.5182	0.3977	0.8109
Significance		*				*		*♣^	♥
MDA (nmol/l)	135.6	120	111.8	127	124	160.3	114.44	131	154
SD	26.416	16.036	28.871	55.794	26.431	72.207	21.066	61.417	35.581
Significance									♠

(FBS) Fasting blood sugar; (HbA1c) glycosylated hemoglobin; (FA) fructosamine; (TAC) total antioxidant capacity; (MDA) malondialdahyde. Symbols represent the followings: (*) statistically significance difference when compared with G1; (♣) statistically significance difference when G5, G6, G7, G8 or G9 compared with G4; (^) statistically significance difference when G8 compared with G6; (♠) statistically significance difference when G9 compared with G7; (♦) statistically significance difference when G7 compared with G6; (♥) statistically significance difference when G9 compared with G8

By comparing G5, G6, G7, G8 and G9 with G4, we found that glucose level is significantly decreased in G5, G7 and G9 (*P* < 0.05) with no significant changes (*P* > 0.05) in G6 and G8 (Fig. 1). In comparison, HbA1c showed non-significant changes in G5, G6, G7, and G8 (*P* > 0.05), but significantly decreased (*P* < 0.05) in G9 (Fig. 2). Fructosamine showed non-significant changes (*P* > 0.05) in G5, G6, G7, and G8, but significantly decreased (*P* < 0.05) in G9 (Fig. 3). TAC is significantly increased (*P* < 0.05) in G8 and showed non-significant changes (*P* > 0.05) in G5, G6, G7 and G9 (Fig. 4). However, there is no significant change in the level of MDA in all groups (Fig. 5).

By comparing G8 with G6 we found that glucose, fructosamine and MDA showed no significant changes, while HbA1c and TAC showed significant increase. By comparing G9 with G7, we found that glucose and fructosamine levels are significantly decreased (*P* < 0.05), while MDA is significantly increased (*P* < 0.05). However, HbA1c and TAC showed non-significant changes (*P* > 0.05). By comparing G7 with G6 we found that glucose is significantly decreased, while HbA1c, fructosamine, TAC and MDA showed non-significant changes. By comparing G9 with G8, we found that glucose and fructosamine levels are significantly decreased (*P* < 0.05), while HbA1c and TAC are significantly decreased (*P* < 0.05). However, MDA showed non-significant change (*P* > 0.05).

### Histopathological findings

**Fig. 6:**
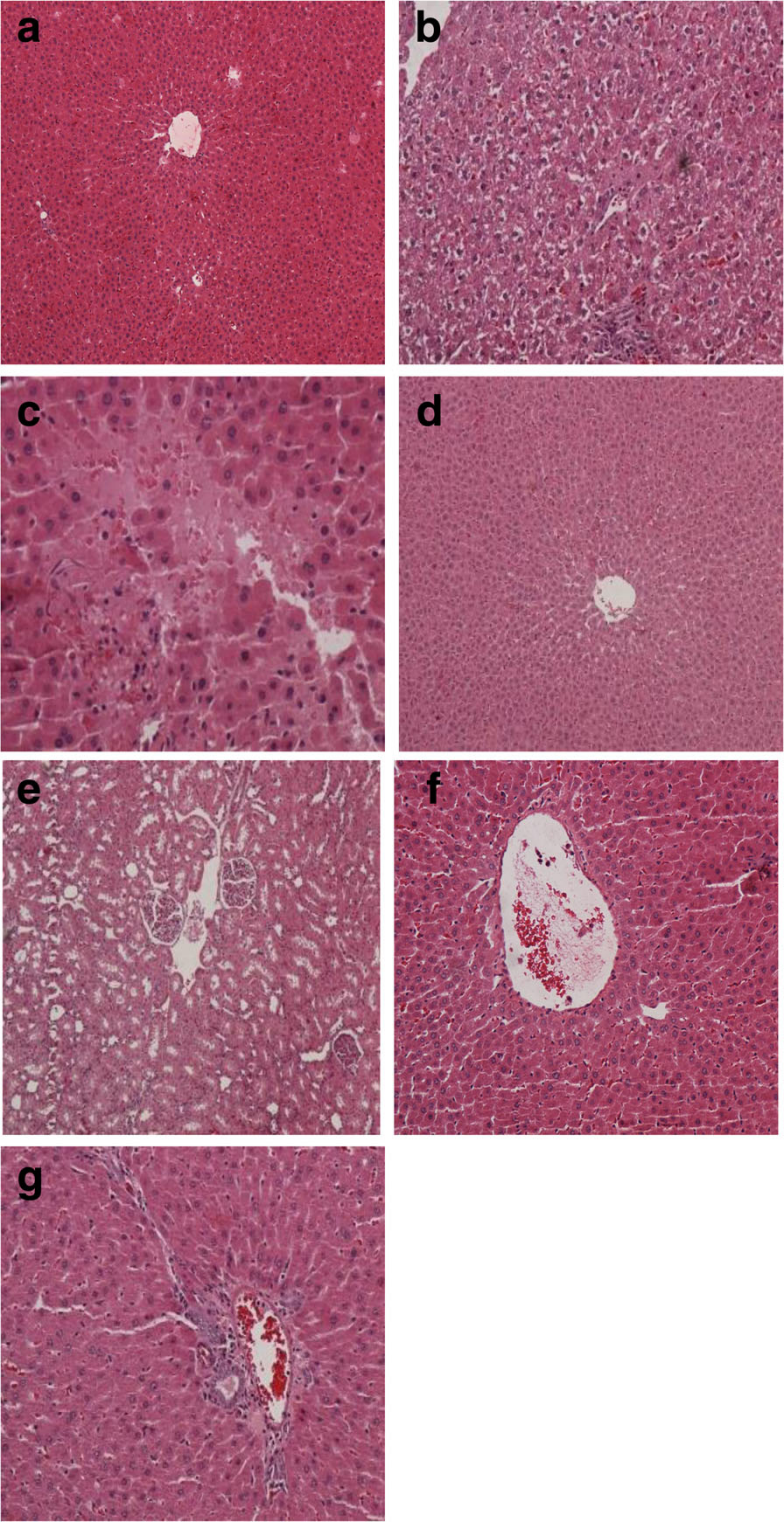
Histopathological examination by light microscope of diabetic rats treated with melatonin or vitamin D in the presence or absence of insulin. Groups are shown as follows: Normal rats (**a**), Diabetic rats (**b**), Diabetic rats treated with insulin (**c**), Diabetic rats treated with melatonin only (**d**), Diabetic rats treated with melatonin and insulin (**e**), Diabetic rats treated with vitamin D only (**f**), Diabetic rats treated with vitamin D and insulin (**g**)

As compared to normal (Fig. 6a), the liver of diabetic rats shows only mild focal microvesicular fatty degeneration (Fig. 6b). The liver of treated diabetic rats with insulin shows degeneration of cell edema in the stroma. The liver exhibited granular degeneration of hepatocytes, including necrosis of individual cells (Fig. 6c). The hepatocytes contained focal fatty vacuoles. The sinusoids are dilated and a progressive loss of general organ structure is seen. Inflammatory changes consistent with steatohepatitis, which are represented by mononuclear inflammatory infiltrates of moderate intensity as observed in periportal spaces. Moreover, the central veins exhibited moderate congestion (Fig. 6c). The liver of diabetic rats treated with melatonin either with insulin or not, exhibited marked improvement (Fig. 6d and e). The liver of diabetic rats taking vitamin D and treated with or without insulin, shows degeneration of cells and edema in the stroma (Fig. 6f and g). The liver exhibited granular degeneration of hepatocytes, including necrosis of individual cells. The hepatocytes contained focal fatty vacuoles. The sinusoids are dilated and a progressive loss of general organ structure is seen. Inflammatory changes are consistent with steatohepatitis, which are represented by mononuclear inflammatory infiltrates of moderate intensity as observed in periportal spaces. Moreover, the central veins exhibited moderate congestion (Fig. 6f and g).

## Discussion

Increased oxidative stress is a widely accepted participant in the development and progression of diabetes and its complications [20–22]. Our results do not show any change in TAC of diabetic rats as compared to normal non-diabetic rats. This may be due to the short period of diabetes induced to rats or to the low glucose concentration in diabetic rats. Strangely enough, our results showed that even in non-treated diabetic rats with insulin, vitamin D administration significantly increased TAC while it is not changed with melatonin either in treated or non-treated groups. This may be explained by the hyperglycemia-induced increase in free radicals, and its impairment of the endogenous antioxidant defense system in many ways during diabetes [23]. Antioxidant defense mechanisms involve both enzymatic and nonenzymatic strategies. Vitamin D alone may have a more significant antioxidant effect on diabetic rats or the concentration of melatonin was insufficient to induce any significant change. It is worth mentioning that the doses selected in our study is according to a pharmacological concept of calculating the animal dose of drugs as 1 to 10 of the human dose per Kg. The duration of the treatment is selected for two reasons: firstly, by comparing the life span of humans, one-month duration of treatment in rat (average life span 2 years) is comparable to 30 months duration treatment of a human average life span of 60 years. Secondly, extending the duration of treatment for more than 1 month leads to more loss and dying of rats.

Despite its discovery over 40 years ago, melatonin was not recognized as a free radical scavenger and antioxidant until the last decade. Prior to that time, the circadian rhythm of melatonin in the blood of mammals was known to be functionally linked to the adjustment of 24-h cycles and to circannual rhythm regulation. Additionally, however, melatonin’s actions include modulation of immune function, tumor growth inhibition and influences on retinal physiology. The free radical scavenging property of melatonin was first suggested by Ianas et al. [24]. Although there have been literally hundreds of publications which demonstrate the free radical scavenging [25–27] and antioxidant actions [28–30] of melatonin both in in vitro and in vivo settings, our results showed that vitamin D is more effective antioxidant and free radical scavenger as illustrated by the significantly increased TAC in diabetic rats that received vitamin D as compared to rats receiving melatonin. Insulin treated diabetic rats and receiving either vitamin D or melatonin are comparable. Therefore, it is evident from our study that the antioxidant capacity and free radical scavenging effects of either melatonin or vitamin D do not depend on controlling the diabetic state and they act as antioxidants whatever the blood glucose level is. However, the dose and duration of melatonin may not be enough.

NAFLD is accompanied by several predisposing, factors such as obesity, diabetes, dyslipidemia, jejunoileal bypass, drugs and parenteral nutrition. Hepatic stellate cells undergo activation, and progression to advanced fibrosis and cirrhosis is possible [31, 32]. Several studies have shown that liver injury in the course of NAFLD is mediated by oxidative stress [33]. Oxidative stress is especially harmful to mitochondria, causing damage that results in impaired gene expression, alterations in proteins synthesis, decreased mitochondrial content and impaired mitochondrial beta-oxidation. Moreover, in the course of NAFLD, mitochondrial CYP2E1 expression increases and causes a redox state [34, 35]. In light of the crucial role of oxidative stress in liver diseases, antioxidants are understandably considered as a good therapeutic strategy for the treatment of liver disorders. To date, the study outcomes remain inconclusive and controversial; however, the therapeutic efficacy of particular antioxidants has been proven [36, 37].

Our study shows that the livers of diabetic rats have only mild focal micro vesicular fatty degeneration but no other abnormalities are observed. Even treated diabetic rats with insulin do not affect liver injury as evidenced by the inflammatory changes consistent with steatohepatitis, which were represented by mononuclear inflammatory infiltrates of moderate intensity, observed in periportal spaces. Melatonin administration to diabetic rats with or without insulin exhibited marked liver improvement. On the other hand, vitamin D administration to diabetic rats treated or not with insulin, do not affect the inflammatory changes of liver cells consistent with steatohepatitis, which are represented by mononuclear inflammatory infiltrates of moderate intensity, observed in periportal spaces.

These discrepancies of our biochemical results to what have been observed in the histopathological studies may be due to the short duration of drug administration to diabetic rats, which may affect either the function of or the liver itself. Despite the numerous studies on humans and animal models, it is extremely difficult to understand and describe the efficacy of antioxidative agents in hepatology [38, 39].

## Conclusion

In conclusion, our results demonstrated the beneficial antioxidant effect of vitamin D administration to normal and diabetic rats as compared to melatonin in our rat model. Nevertheless, still melatonin shows more therapeutic effect on liver cell injury induced by induction of diabetes.

## Abbreviations

DM: Diabetes mellitus
DS: Standard deviation
EDTA: Ethylene diamine tetra acetic acid
FA: Fructosamine
FBS: Fasting blood sugar
HbA1c: Glycosylated hemoglobin
MDA: Malondialdahyde
NAFLD: Non-alcoholic fatty liver disease
ROS: Reactive oxygen species
STZ: Streptozotocin
T1D: Type 1 diabetes
T2D: Type 2 diabetes
TAC: Total antioxidant capacity

## References

[1] Wild S, Roglic G, Green A, Sicree R, King H (2004). Global prevalence of diabetes: estimates for the year 2000 and projections for 2030. Diabetes Care.

[2] Matheus AS, Tannus LR, Cobas RA, Palma CC, Negrato CA, Gomes MB (2013). Impact of diabetes on cardiovascular disease: an update. Int J Hypertens.

[3] Zhang JJ, Meng X, Li Y, Zhou Y, Xu DP, Li S, Li HB. Effects of Melatonin on Liver Injuries and Diseases. Int J Mol Sci. 2017;18(4). doi:10.3390/ijms18040673. 10.3390/ijms18040673 PMC5412268 28333073

[4] Cusi K (2009). Nonalcoholic fatty liver disease in type 2 diabetes mellitus. Curr Opin Endocrinol Diabetes Obes.

[5] Chavan M, G S, G RR, Eage P: Prevalence of fatty liver disease among type-2 diabetes mellitus patients and its relation to insulin resistance. J Assoc Physicians India 2016, 64(1):51.

[6] Guven A, Yavuz O, Cam M, Ercan F, Bukan N, Comunoglu C, Gokce F (2006). Effects of melatonin on streptozotocin-induced diabetic liver injury in rats. Acta Histochem.

[7] Nannipieri M, Gonzales C, Baldi S, Posadas R, Williams K, Haffner SM, Stern MP, Ferrannini E (2005). Mexico City diabetes s: Liver enzymes, the metabolic syndrome, and incident diabetes: the Mexico City diabetes study. Diabetes Care.

[8] Mohamed J, Nazratun Nafizah AH, Zariyantey AH, Budin SB (2016). Mechanisms of Diabetes-Induced Liver Damage: The role of oxidative stress and inflammation. Sultan Qaboos Univ Med J.

[9] Reiter RJ, Tan DX, Burkhardt S (2002). Reactive oxygen and nitrogen species and cellular and organismal decline: amelioration with melatonin. Mech Ageing Dev.

[10] Wei Y, Chen P, de Bruyn M, Zhang W, Bremer E, Helfrich W (2010). Carbon monoxide-releasing molecule-2 (CORM-2) attenuates acute hepatic ischemia reperfusion injury in rats. BMC Gastroenterol.

[11] Parveen K, Khan MR, Mujeeb M, Siddiqui WA (2010). Protective effects of Pycnogenol on hyperglycemia-induced oxidative damage in the liver of type 2 diabetic rats. Chem Biol Interact.

[12] Cahova M, Palenickova E, Dankova H, Sticova E, Burian M, Drahota Z, Cervinkova Z, Kucera O, Gladkova C, Stopka P (2015). Metformin prevents ischemia reperfusion-induced oxidative stress in the fatty liver by attenuation of reactive oxygen species formation. Am J Physiol Gastrointest Liver Physiol.

[13] Korkmaz GG, Uzun H, Cakatay U, Aydin S (2012). Melatonin ameliorates oxidative damage in hyperglycemia-induced liver injury. Clin Invest Med.

[14] Tahan G, Gramignoli R, Marongiu F, Aktolga S, Cetinkaya A, Tahan V, Dorko K (2011). Melatonin expresses powerful anti-inflammatory and antioxidant activities resulting in complete improvement of acetic-acid-induced colitis in rats. Dig Dis Sci.

[15] Agil A, El-Hammadi M, Jimenez-Aranda A, Tassi M, Abdo W, Fernandez-Vazquez G, Reiter RJ (2015). Melatonin reduces hepatic mitochondrial dysfunction in diabetic obese rats. J Pineal Res.

[16] Nakashima A, Yokoyama K, Yokoo T, Urashima M (2016). Role of vitamin D in diabetes mellitus and chronic kidney disease. World J Diabetes.

[17] Barchetta I, Del Ben M, Angelico F, Di Martino M, Fraioli A, La Torre G, Saulle R, Perri L, Morini S, Tiberti C (2016). No effects of oral vitamin D supplementation on non-alcoholic fatty liver disease in patients with type 2 diabetes: a randomized, double-blind, placebo-controlled trial. BMC Med.

[18] Liu L, Lv G, Ning C, Yang YE, Zhu J (2016). Therapeutic effects of 1,25-dihydroxyvitamin D3 on diabetes-induced liver complications in a rat model. Exp Ther Med.

[19] Masiello P, Broca C, Gross R, Roye M, Manteghetti M, Hillaire-Buys D, Novelli M, Ribes G (1998). Experimental NIDDM: development of a new model in adult rats administered streptozotocin and nicotinamide. Diabetes.

[20] Ceriello A (2000). Oxidative stress and glycemic regulation. Metabolism.

[21] Baynes JW, Thorpe SR (1999). Role of oxidative stress in diabetic complications: a new perspective on an old paradigm. Diabetes.

[22] Baynes JW (1991). Role of oxidative stress in development of complications in diabetes. Diabetes.

[23] Saxena AK, Srivastava P, Kale RK, Baquer NZ: Impaired antioxidant status in diabetic rat liver. Effect Vanadate *Biochem Pharmacol* 1993, 45(3):539-542. 10.1016/0006-2952(93)90124-f 8442752

[24] Ianas O, Olinescu R, Badescu I (1991). Melatonin involvement in oxidative processes. Endocrinologie.

[25] Hardeland R, Balzer I, Poeggeler B, Fuhrberg B, Uria H, Behrmann G, Wolf R, Meyer TJ, Reiter RJ (1995). On the primary functions of melatonin in evolution: mediation of photoperiodic signals in a unicell, photooxidation, and scavenging of free radicals. J Pineal Res.

[26] Reiter RJ, Tan DX, Manchester LC, Qi W (2001). Biochemical reactivity of melatonin with reactive oxygen and nitrogen species: a review of the evidence. Cell Biochem Biophys.

[27] Tan DX, Manchester LC, Reiter RJ, Qi WB, Karbownik M, Calvo JR (2000). Significance of melatonin in antioxidative defense system: reactions and products. Biol Signals Recept.

[28] Pappolla MA, Chyan YJ, Poeggeler B, Frangione B, Wilson G, Ghiso J, Reiter RJ (2000). An assessment of the antioxidant and the antiamyloidogenic properties of melatonin: implications for Alzheimer's disease. J Neural Transm (Vienna).

[29] Reiter RJ (1998). Oxidative damage in the central nervous system: protection by melatonin. Prog Neurobiol.

[30] Reiter RJ, Carneiro RC, Oh CS (1997). Melatonin in relation to cellular antioxidative defense mechanisms. Horm Metab Res.

[31] Musso G, Gambino R, Cassader M (2010). Non-alcoholic fatty liver disease from pathogenesis to management: an update. Obes Rev.

[32] Koek GH, Liedorp PR, Bast A (2011). The role of oxidative stress in non-alcoholic steatohepatitis. Clin Chim Acta.

[33] Matthew Morris E, Fletcher JA, Thyfault JP, Rector RS (2013). The role of angiotensin II in nonalcoholic steatohepatitis. Mol Cell Endocrinol.

[34] Santos JC, Valentim IB, de Araujo OR, Ataide Tda R, Goulart MO (2013). Development of nonalcoholic hepatopathy: contributions of oxidative stress and advanced glycation end products. Int J Mol Sci.

[35] Kathirvel E, Chen P, Morgan K, French SW, Morgan TR (2010). Oxidative stress and regulation of anti-oxidant enzymes in cytochrome P4502E1 transgenic mouse model of non-alcoholic fatty liver. J Gastroenterol Hepatol.

[36] Esrefoglu M (2012). Oxidative stress and benefits of antioxidant agents in acute and chronic hepatitis. Hepat Mon.

[37] Ha HL, Shin HJ, Feitelson MA, Yu DY (2010). Oxidative stress and antioxidants in hepatic pathogenesis. World J Gastroenterol.

[38] Al-Busafi SA, Bhat M, Wong P, Ghali P, Deschenes M (2012). Antioxidant therapy in nonalcoholic steatohepatitis. Hepat Res Treat.

[39] Singal AK, Jampana SC, Weinman SA (2011). Antioxidants as therapeutic agents for liver disease. Liver Int.

